# The impact of global recruitment and country income on placebo rates in inflammatory bowel disease clinical trials

**DOI:** 10.1093/ecco-jcc/jjag081

**Published:** 2026-06-25

**Authors:** Shane W Goodwin, Yuhong Yuan, Siddharth Singh, Virginia Solitano, Vineet Ahuja, Sudheer Kumar Vuyyuru, Mohammad Shehab, Neeraj Narula, Christopher Ma, Vipul Jairath

**Affiliations:** Department of Medicine, London Health Science Centre Research Institute, London Health Science Centre, London, ON, Canada; Department of Medicine, London Health Science Centre Research Institute, London Health Science Centre, London, ON, Canada; Department of Medicine, Western University, London, ON, Canada; Division of Gastroenterology and Hepatology, Department of Medicine, Mayo Clinic Arizona, Scottsdale, AZ, United States; Division of Gastroenterology and Gastrointestinal Endoscopy, IRCCS Ospedale San Raffaele, Università Vita-Salute San Raffaele, Milan, Italy; Department of Epidemiology and Biostatistics, Western University, London, ON, Canada; Department of Gastroenterology, All India Institute of Medical Sciences, New Delhi, India; Department of Medicine, Western University, London, ON, Canada; Program of Medicine, College of Medicine and Health Sciences, Abdullah Al Salem University, Khaldiya, Kuwait; Division of Gastroenterology and Hepatology, McGill University Health Center, Montreal, QC, Canada; Department of Medicine (Division of Gastroenterology) and Farncombe Family Digestive Health Research Institute, McMaster University, Hamilton, ON, Canada; Department of Community Health Sciences, Cumming School of Medicine, University of Calgary, Calgary, AB, Canada; Division of Gastroenterology and Hepatology, Department of Medicine, University of Calgary, Calgary, AB, Canada; Department of Medicine, Western University, London, ON, Canada; Department of Epidemiology and Biostatistics, Western University, London, ON, Canada

**Keywords:** placebo response, Crohn’s disease, ulcerative colitis, inflammatory bowel disease, study design

## Abstract

**Introduction:**

Despite progress in understanding placebo effects in clinical trials of inflammatory bowel disease (IBD), there is a limited understanding of how socioeconomic factors of recruiting countries impact placebo rates. We investigated the association of socioeconomic factors across recruitment sites on placebo rates in IBD randomized controlled trials.

**Methods:**

We examined the association of clinical and endoscopic remission and response to the weighted Gross National Index (GNI) per capita, Human Development Index (HDI), and out-of-pocket health expenditures as a percentage of current health expenditures (OOP_CHE) of identified trials using mixed effects linear regression.

**Results:**

Across 76 trials (Crohn’s disease [CD]: *n* = 32; ulcerative colitis [UC]: *n* = 44), placebo response rates over time were largely stable, with a small increase across time in clinical remission for CD trials (β = 0.4% per increase in study year, 95% CI: 0.3%-0.84%, *P* = .04) and a small decrease across time in endoscopic response for UC trials (β = −1.39% per increase in study year, 95% CI: −1.79, −0.99, *P* < .001). Clinical response was most susceptible to changes in socioeconomic factors in IBD trials (β = −2.96% per 10 000 USD increase in study GNI, 95% CI: −4.98, −0.95%, *P* = .004; *P* < .05 for HDI and OOP_CHE). Across both conditions, decreases in clinical response were associated with increases in HDI and OOP_CHE (CD HDI: β = −1.15% per 0.01 increase in study HDI, 95% CI: −2.24, −0.06%, *P* = .04; UC HDI: β = −0.66% per 0.01 increase in study HDI, 95% CI: −1.19, −0.12%, *P* = .02).

**Discussion:**

We found that placebo clinical response was most susceptible to influences of site-level socioeconomic wellbeing. These findings highlight that estimation of placebo rates in trial planning should account for geographic location of recruiting sites.

## 1. Introduction

As placebo-controlled trials are the gold standard in study design, identification of risk factors associated with placebo response is a critical aspect of clinical trial design.^1^ In inflammatory bowel disease (IBD), recent studies have suggested risk factors related to disease phenotype and clinical characteristics.^2–7^ While our understanding of patient characteristics continues to grow, there is limited knowledge regarding how site recruitment factors may influence study outcomes. This is important to understand since the globalization of IBD as a disease has also resulted in the opening of clinical trial sites in nontraditional jurisdictions. A 2025 study by Kerschbaumer et al. in psoriatic arthritis reported increasing placebo response rates in clinical trials over time, proposing that this may be driven by increased global recruiting patterns, with increasing inclusion of less affluent countries in trial recruitment.^8^ These findings were further confirmed in separate studies of trials in rheumatoid arthritis^9^ and focal epilepsy.^10^

For IBD, no study has examined whether placebo outcomes are changing over time, and whether this could be influenced by the affluence of recruiting countries. While individual patient and disease characteristics are most likely to influence placebo rates, certain socioeconomic factors such as the overall economic status of a country, out-of-pocket expenditures, or broader indicators of social inequality may also impact these rates.^11^ In this ecological study, we examined whether placebo outcomes in IBD clinical trials are changing across time and whether these changes are impacted by study-level differences in country-averaged socioeconomic factors.

## 2. Methods

### 2.1. Search strategy and data extraction

We searched MEDLINE, EMBASE, and the Cochrane Central Register of Controlled Trials (CENTRAL) (all via Ovid) from 2023 to March 17, 2025 to identify English-language, phase 2 and phase 3 placebo-controlled randomized controlled trials in IBD. Both controlled vocabulary (eg, MeSH and Emtree terms) and free-text keywords related to IBD, ulcerative colitis (UC) and Crohn’s disease (CD) were searched. Studies published before 2023 were identified through a review of reference lists from two recently published systematic reviews and network meta-analyses.^12–13^

When National Clinical Trial (NCT) numbers were available for trials registered on ClinicalTrials.gov, we examined the corresponding study protocols for additional information. From each trial, the number of recruiting sites per country was extracted, along with information on the study start year, placebo sample size, and the number of patients displaying placebo response outcomes. Outcomes collected were clinical remission, clinical response, endoscopic remission, and endoscopic response.

### 2.2. Outcome definitions

Studies varied in their definitions and collection times for each definition, reflecting the diversity in outcome measurement in the IBD field. In CD trials, clinical remission was measured using a definition of Crohn’s Disease Activity Index (CDAI) score < 150 in all studies except one, where clinical remission was measured using a reduction of 30% in either stool frequency or abdominal pain scores. Clinical response was consistently measured by a decrease in CDAI scores of at least 70-100 points. Most CD-measurement variability was in the timing of measurement for clinical remission and response ranging from examining outcomes at 4-16 weeks. For UC trials, clinical remission was measured most often using a definition of Mayo Clinical Score < 3 with no subscore > 1 or using the Adapted Mayo Score < 3 with stool frequency score < 1 and not greater than baseline. Additionally, the majority of studies required a rectal bleeding score of 0. Endoscopic outcomes were assessed using the Mayo Endoscopic Subscore. As with CD trials, the majority of measurement variability was in the timing, with outcomes measured between weeks 6 and 14.

Following previous research,^8–10^ the number of recruitment sites for each country was extracted for each included clinical trial. This approach was validated by Kerschbaumer et al. by showing a strong association between the number of recruitment sites in a country and the number of patients per country included in the trial.^8^ Site recruitment was extracted from the publication or the published study protocol, and in cases where the study protocol was unavailable, this information was extracted from https://clinicaltrials.gov.

### 2.3. Assessment of socio-economic status of sites

Three factors were used to examine recruitment-site socioeconomic influences on study outcomes, each modeled separately to avoid risk of collinearity among the indicators. As the timing of inclusion of a recruitment site was not reported, each of the three study-level characteristics were based on the starting recruitment year. First, Gross National Index (GNI) per capita was used as the primary proxy for recruitment site affluence, and the average GNI of each study was weighted by the proportion of recruitment sites across different countries included in the study. Information on whether a site was successful in recruiting a patient was unavailable for all studies, and as such we assume the distribution of patients matches the distribution of recruitment sites. GNI country-level data were obtained from World Bank^14^ and GNI was calculated using the Atlas method and reported in 2024 USD to aid in comparisons across studies.

Second, the Human Development Index (HDI) was included as a broad social indicator obtained from the United Nations Development Programme to capture factors influencing health.^15^ The HDI incorporates factors such as life expectancy at birth and the mean years of schooling, along with GNI to provide a broad perspective on country-level socioeconomic well-being and ranges from 0 to 1, with values closer to 1 indicating better development (life expectancy of 85+ years, 18 years of schooling, and a GNI per capita of $75 000).^15^

Third, out-of-pocket-expenditures as a percentage of current health expenditures (OOP_CHE) was used as an indicator of access to healthcare. OOP_CHE was obtained from the World Health Organization and includes expenses such as consultations, medical treatment, and hospital care, but does not include insurance premiums, tax-based health services, or employer contributions.^16^ The OOP_CHE is reported as a percentage of the total country-level health spending, with higher percentages associated with less access to care. For a sensitivity analysis of more recent studies (2010 onwards), we also included the World Health Organizations Income Inequality indicator based on the Gini coefficient, providing an estimate of the inequality in the distribution of income within a country.^16^ As recruited trials span approximately 20 years, to reduce confounding of longitudinal impacts on socioeconomic indicators, each indicator was normalized by the mean of the indicator for the including studies, as described by Kerschbaumer et al.^8^

### 2.4. Data analysis

Dot plots were produced to descriptively examine trends in placebo outcomes across time and for each normalized indicator (GNI, HDI, OOP_CHE), where each dot represents a specific study and the size of a study represents the number of recruitment sites included in the trial. Mixed effect linear regression models with a random intercept for trial study were used to examine differences in placebo response outcomes across time and by the normalized indicator. CD and UC trials were examined separately. Due to the small number of trials for each condition, a sensitivity analysis combining CD and UC trials was examined. Additional sensitivity analyses included the possible impact of a study receiving central reading vs local reading may have on outcome and the timing of induction. As not all studies explicitly indicated if central reading occurred, we compared an indicator of those studies in 2016 and onwards compared to earlier studies, as by 2016 the majority of studies were conducting central reading. All analyses were constructed using R v.4.3.1.^17^

## 3. Results

**Figure 1.**
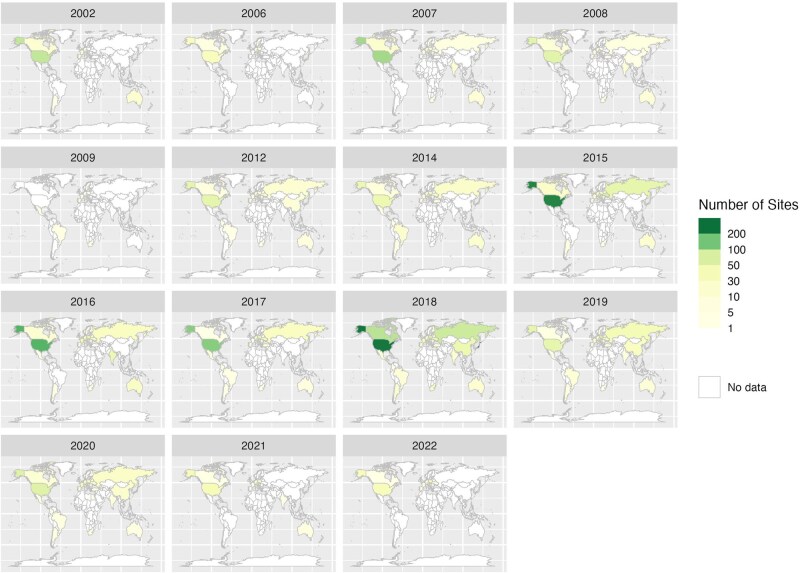
Global recruitment patterns in phase 2 and 3 placebo controlled clinical trials for ulcerative colitis.

**Figure 2.**
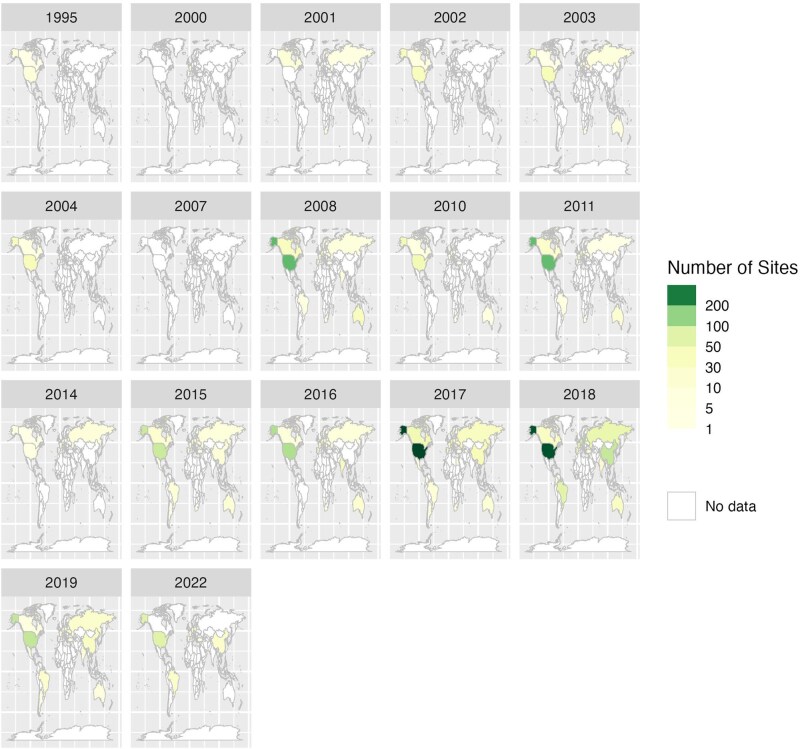
Global recruitment patterns in phase 2 and 3 placebo controlled clinical trials for Crohn’s disease.

A total of 77 phase 2 or 3 placebo-controlled trials were identified containing sufficient site recruitment data (CD: *n* = 32; UC: *n* = 44), with trial start dates ranging from 1995 to 2022 in CD trials and from 2002 to 2022 for UC trials. One trial for UC was identified but excluded due to lack of site recruitment information, leaving a total of 76 trials available for analysis. A visual inspection of site recruitment over time suggests an increase in the number of countries and recruitment sites included in each study was observed (see Figures 1 and 2). Across both conditions, site recruitment expanded from North America and Western Europe to include larger numbers of East Europe and East Asian countries across time, with site recruitment peaking around 2018.

Normalized estimates ranged from $9854 to $55 047 (nGNI), 0.773 to 0.934 (nHDI), and 9.45% to 35.14% (nOOP_CHE) for CD trials, and from $7876 to $52 826 (nGNI), 0.751 to 0.988 (nHDI), and 13.33% to 38.47% (nOOP_CHE) for UC trials. Linear regression analysis suggests a negative association of nGNI per study with the year of study conduction, with more recent studies displaying a lower nGNI due to an increase in the proportion of included affluent ­countries (mean change in nGNI per study year: −373.4 USD, 95% CI: −687.74, −59.10, *P* = .02, see Figure S1).

### 3.1. Placebo response across time

**Figure 3.**
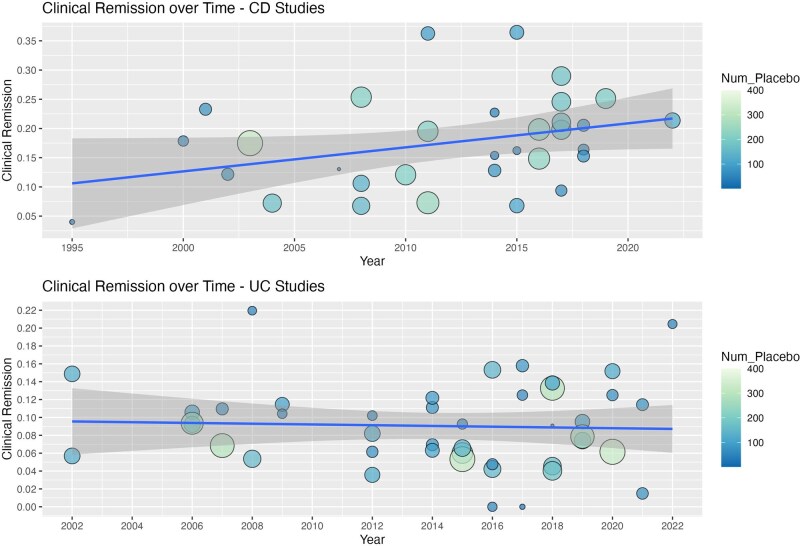
Placebo response clinical remission across time in Crohn’s disease and ulcerative colitis trials.

In CD trials, mixed effect models found a positive association of placebo response rates with the study start year for outcomes of clinical remission (β = 0.4% per increase in study year, 95% CI: 0.3%-0.84%, *P* = .04, Figure 3). No statistical association was found for clinical response (*P* = .08), endoscopic remission (*P* = .18), or endoscopic response (*P* = .95) (see Figures S2–S4).

In UC trials, mixed effect models found a decrease in endoscopic response across time (β = −1.39% per increase in study year, 95% CI: −1.79, −0.99, *P* < .001), with no statistical association for clinical remission (*P* = .49), clinical response (*P* = .34), or endoscopic remission (*P* = .82) across time (see Figures S2–S4).

### 3.2. Placebo response and socioeconomic well-being

**Figure 4.**
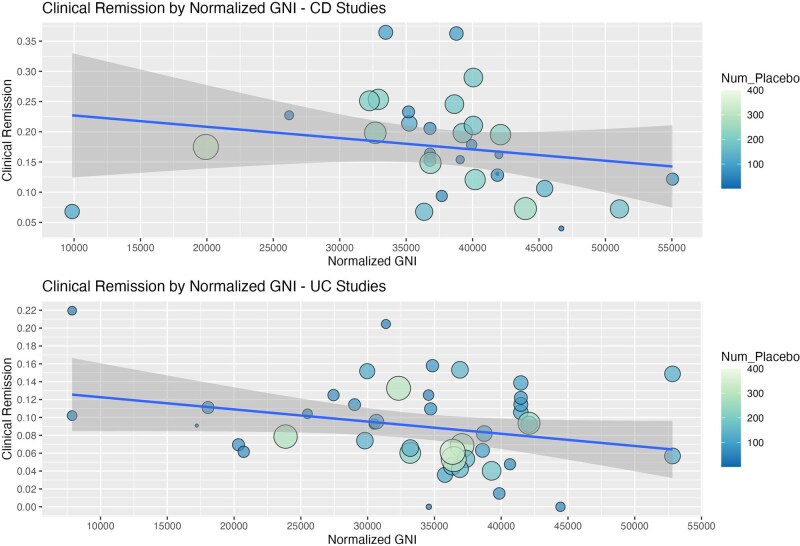
Placebo response clinical remission rates by normalized Gross National Index (GNI) per capita in phase 2 and 3 clinical trials for Crohn’s disease and ulcerative colitis trials.

**Figure 5.**
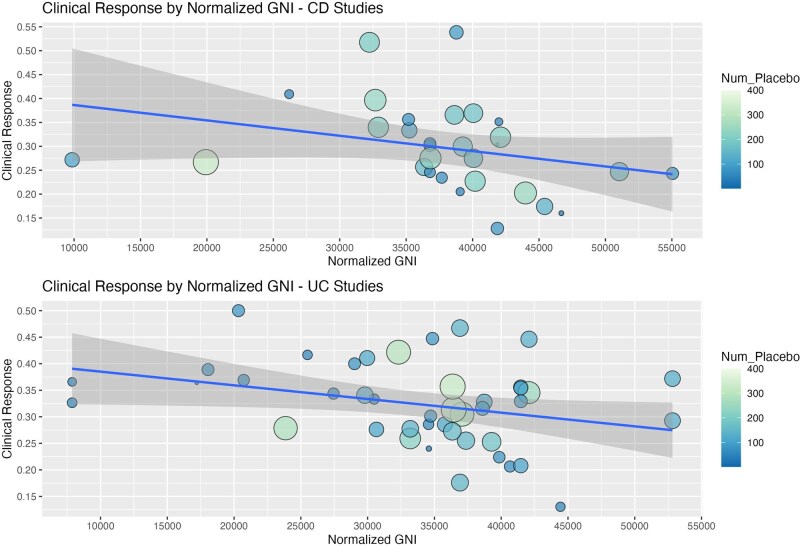
Placebo clinical response by normalized Gross National Index (GNI) per capita in phase 2 and 3 clinical trials for Crohn’s disease and ulcerative colitis trials.

In CD trials, mixed effect models found a significant decrease in endoscopic response associated with an increase in weighted normalized study GNI (β = −7.61% per 10 000 USD increase in study GNI, 95% CI: −14.7, −0.53%, *P* = .04) and a significant decrease in clinical response associated with an increase in HDI (β = −1.15% per 0.01 increase in study HDI, 95% CI: −2.24, −0.06%, *P* = .04). For GNI, no statistical association was found for clinical remission (*P* = .34), clinical response (*P* = .13), or endoscopic remission (*P* = .46), while for HDI, no statistical association was found for clinical remission (*P* = .46), endoscopic remission (*P* = .10), or endoscopic response (*P* = .12). No statistical association was found between OOP_CHE and placebo response (*P* > .05 for each outcome). See Figures 4 and 5 and Figures S5–S8 for full data visualizations.

In UC trials, mixed effect models found a significant decrease in endoscopic remission associated with an increase in weighted normalized study GNI (β = −1.72% per 10 000 USD increase in study GNI, 95% CI: −2.8, −0.57%, *P* = .003) and a significant decrease in clinical response associated with an increase in HDI (β = −0.66% per 0.01 increase in study HDI, 95% CI: −1.19, −0.12%, *P* = .02) and an increase in OOP_CHE (β = 0.48% per one per cent increase in study OOP_CHE, 95% CI: 0.1, 0.86%, *P* = .02). For GNI, no statistical association was found for clinical remission (*P* = .13), clinical response (0.07), or endoscopic response (*P* = .45), while for HDI and OOP_CHE, no statistical association was found for clinical remission (*P* = .26 and *P* = .31), endoscopic remission (*P* = .06 and *P* = .051), or endoscopic response (*P* = .79 and *P* = .15).

### 3.3. Sensitivity analyses

Using both CD and UC trials combined, mixed effect models found a decrease in clinical response (β = −2.96% per 10 000 USD increase in study GNI, 95% CI: −4.98, −0.95%, *P* = .004), and non-significant results for outcomes of clinical remission (*P* = .63), endoscopic remission (*P* = .06), and endoscopic response (*P* = .73). Similar results were found using normalized HDI (*P* > .05 for all outcomes, except clinical response: β = −0.63% per 0.01 increase in study HDI, 95% CI: −1.13, −0.12%, *P* = .02) and OOP_CHE (*P* > .05 for all outcomes, except clinical response: β = 0.45% per one per cent increase in study OOP_CHE, 95% CI: 0.09, 0.8%, *P* = .01).

Additionally, using an indicator of income inequality across included countries from 2010 onwards, no statistical association between any outcome was identified (*P* > .05 for each). This held true regardless of whether CD and UC were examined independently or combined.

The inclusion of a central reading indicator found no statistical significance across any outcome or in models with socioeconomic adjustment, except for endoscopic response over time in UC studies (*P* < .05). However, once adjusted for temporal effects, the central reading indicator became statistically non-significant.

The inclusion of timing of induction impacted temporal associations for clinical remission and response but had no impact on endoscopic response, nor associations with socioeconomic status variables. Specifically, in patients with CD, the temporal association identified previously became non-significant, changing from β = 0.4% per increase in study year to −0.03% (95% CI: −0.4, 0.4%, *P* = .83), and in UC patients, the temporal association for clinical remission and clinical response became statistically significant, β = −0.3% (95% CI: −0.58, −0.07, *P* = .01) and β = −0.6% (95% CI: −1.1, −0.04, *P* = .04). No changes in inclusion of induction timing were found for other outcomes or in models that included socioeconomic indicators.

## 4. Discussion

In this ecological study, we identified that over 25 years, placebo rates in phase 2 and 3 placebo-controlled UC trials have been largely stable while rates have been slowly increasing in CD trials. Visually, IBD trial data were aligned with effects reported in the literature regarding the association between placebo rates and country affluence, particularly for the endpoints of clinical remission and clinical response.^8–10^ However, indicators of socioeconomic well-being were not always consistent in statistical significance, for example in CD trials where normalized GNI is a statistically associated factor for endoscopic response while HDI and OOP_CHE were found to be associated with clinical response. However, HDI and OOP_CHE were consistent in their association with clinical response, and furthermore, when CD and UC data were combined to increase the number of studies, all three indicators consistently were associated with changes in clinical response. Taken as a whole, our study suggests that clinical response is most sensitive to recruitment site affluence.

There were some unexpected findings. In CD trials, a positive association between normalized GNI and endoscopic response was identified, and a similar association between normalized GNI and endoscopic remission was identified in UC trials. In both cases, we expect that these associations may be a spurious finding. In both cases very few trials provided endoscopic information and given the lack of consistency across indicators, nor when CD and UC studies were analyzed together, we believe this association is likely to be an artifact of the data and should be interpreted cautiously. A second unexpected finding was the lack of statistical association between clinical remission and any of the socioeconomic indicators in both CD and UC trials. The normalization of GNI across IBD trials restricted the variation in the study weighted GNI and this lack of variation may explain the non-significant effects. When clinical remission and response were examined using non-normalized study weighted GNI, both outcomes were statistically associated with GNI, highlighting the reduced variation witnessed by removing the temporal confounding associated with GNI and time.

While our results may not perfectly align with previous research, it may be a consequence of the outcomes used in IBD trials compared to other research fields. For example, it is important to highlight that in the previous literature, such as reported by Kerschbaumer et al. in 2025, the outcomes examined were closer in alignment to our outcome of clinical response rather than remission.^8^ It is likely that the impact of site recruitment affluence is small, such that only outcomes with larger trial variation are most susceptible.

We believe our results are aligned with the theoretical structure of clinical and endoscopic outcomes. As endoscopic remission and response are based on objective measures of disease activity, we expected and our results suggest that subjective measures of disease activity are more vulnerable to placebo effects. A previous study suggested the subjective components of the CDAI are most susceptible to variability and it is plausible that these components drive the significant association between GNI and placebo outcomes.^18^ The causal association between socioeconomic well-being and placebo response rates is not well understood, with hypothesized pathways relating to health care and medical access being the primary motivators for trial enrollment and trial continuation.^8–10^^,^^19^^,^^20^ Previous research suggests that access to treatments and treatment reimbursement are highest in Western and Northern European countries, reflecting the lower out-of-pocket expenditures as a percentage of current health expenditures and the overall higher affluence of these countries.^21^ However, it should be stressed that the effect of socioeconomic factors across trials was small, suggesting the affluence of recruitment sites represent only one factor in differences in placebo response rates across studies, and other factors previously suggested by studies are at least as equally important.^2–7^

Our findings reflect the changing recruitment patterns and observation that study-average GNI is increasing across time. While recent studies have included more emergent economy countries, the inclusion of additional European countries and Scandinavian recruitment sites have aided in the increase in study-average GNI across time. Recruitment to IBD clinical trials continue to be North American and Europe dominant.^22^ With the globalization of IBD and need for more diverse and efficient recruitment, there are perceived barriers in launching trials in new jurisdictions due to differences in standards of care, cultural differences in symptom reporting, regulatory environment, and site-level infrastructure.^23^ However, it is essential to address these perceived barriers as emerging economies make up the majority of the world’s population and with IBD becoming a global disease, emergent economies will continue to make up a larger percentage of recruiting sites across time.^23–24^ Calls for improved access and quality of health care provided in Eastern Europe and emergent economies will continue to increase placing additional pressure to diversify patient populations in IBD clinical trials.^24^ As such, it is imperative we continue to understand the ways study recruitment and geographic location may influence study outcomes.

There are several limitations of this study. The number of studies available for inclusion was relatively small, limiting statistical inference, especially for endoscopic outcomes, in which nearly half of studies did not report this. Small samples limited the ability to control other study characteristics, and findings should be interpreted as a first exploratory step. Sample size limited exploration of bio-naive status, an important indicator that may be associated with placebo response rates, as noted in the work by Lee et al.^25^ If bio-naive patients are more likely to achieve clinical outcomes and patients in low socioeconomic countries are more likely to be bio-naive then any identified effects may be attributed to their prior treatment status rather than socioeconomic position. Further work to elucidate the naive-status of patients would better clarify this point. Additionally, we used reported recruitment sites as a proxy for the location of placebo patients; however, it is unknown which sites actually contributed to the number of placebo patients. However, previous research validating the approach of using recruitment-site information as a proxy for the number of patients included in a study suggests validity of the approach.^8^ It would be beneficial for future studies to report detailed patient information related to the country of recruitment to better connect outcomes to recruitment locations. Related to this point, there is an inherent assumption in this approach that no regional differences exist across socioeconomic indicators. This is probably not the case and certain regions are more likely to deviate from the country-level average. Without access to patient-level information it is difficult to adjust for regional effects and the strength of any regional effects may impact our findings. Additionally, there is the possibility that central versus local reading may impact study outcomes and our exploration of this was limited due to lack of detailed information across all studies. We have used 2016 as a proxy indicator for central reading and while there was no statistical effect there is the possibility that a more refined indicator variable may capture any central reading effects. Finally, it is unknown whether other measures of affluence would better capture differences in placebo outcomes across studies, particularly having access to individual-level information on socioeconomic status. As we are unable to attribute a specific placebo response patient to a location, we are only able to apply a study-averaged estimate of affluence to a particular trial placebo response rate, and this may not accurately reflect the true association. It would be beneficial for future clinical trials in IBD to validate this approach.

Overall, we found preliminary evidence of the positive association between decreasing study socioeconomic well-being and increases in placebo response rates, with a stronger association for clinical response compared to other outcomes. This study highlights how study characteristics related to the sociodemographic factors of a recruitment location may impact placebo rates. Investigators should be aware of the impact that a recruitment site location may have on placebo response rates during trial design and consider ways to handle patient expectations such that motivating factors for placebo responses are minimized, regardless of site location. This may include trial design strategies such as site-stratified randomization or use of patient expectation surveys to aim at reducing placebo responses, particularly at those locations more vulnerable to affluence-based increased placebo response. Future trials should collect and publish additional geographic information surrounding recruitment sites such that socioeconomic assessments can be conducted and compared across treatment and placebo groups to future enhance our understanding of design-based influences on study outcomes.

## Supplementary Material

jjag081_Supplementary_Data

## Data Availability

The data presented in this study may be available on request from the corresponding author.
