# Supplementary figures and images for: The impact of global recruitment and country income on placebo rates in inflammatory bowel disease clinical trials
Source: J Crohns Colitis. 2026 Jun 25;20(6):jjag081. doi: 10.1093/ecco-jcc/jjag081 (PMC13303291; doi:10.1093/ecco-jcc/jjag081)

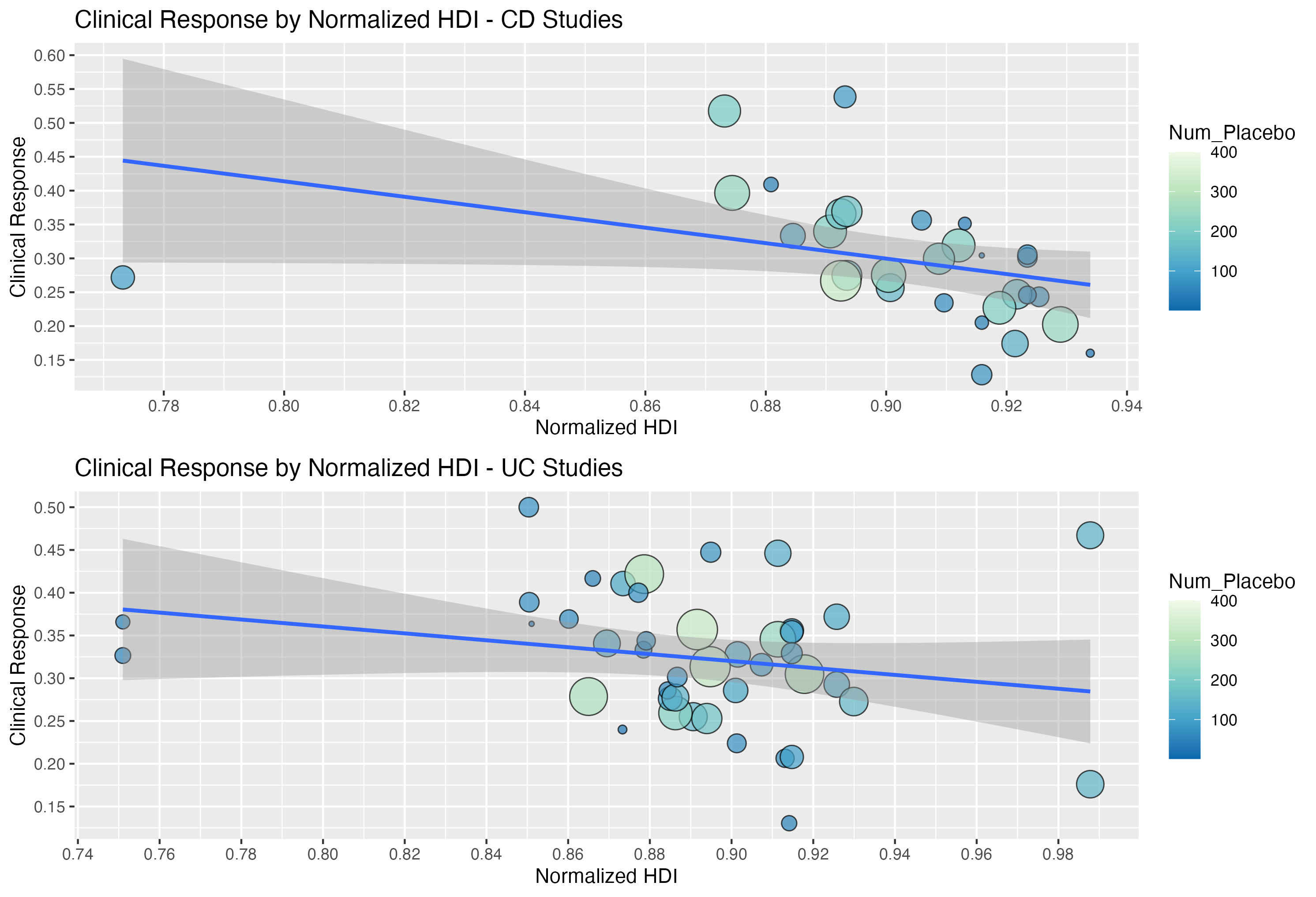

Supplement: jjag081_Supplementary_Data [file jjag081_supplementary_data.zip › Figure S7 - Clinical Response by Normalized HDI.jpeg]

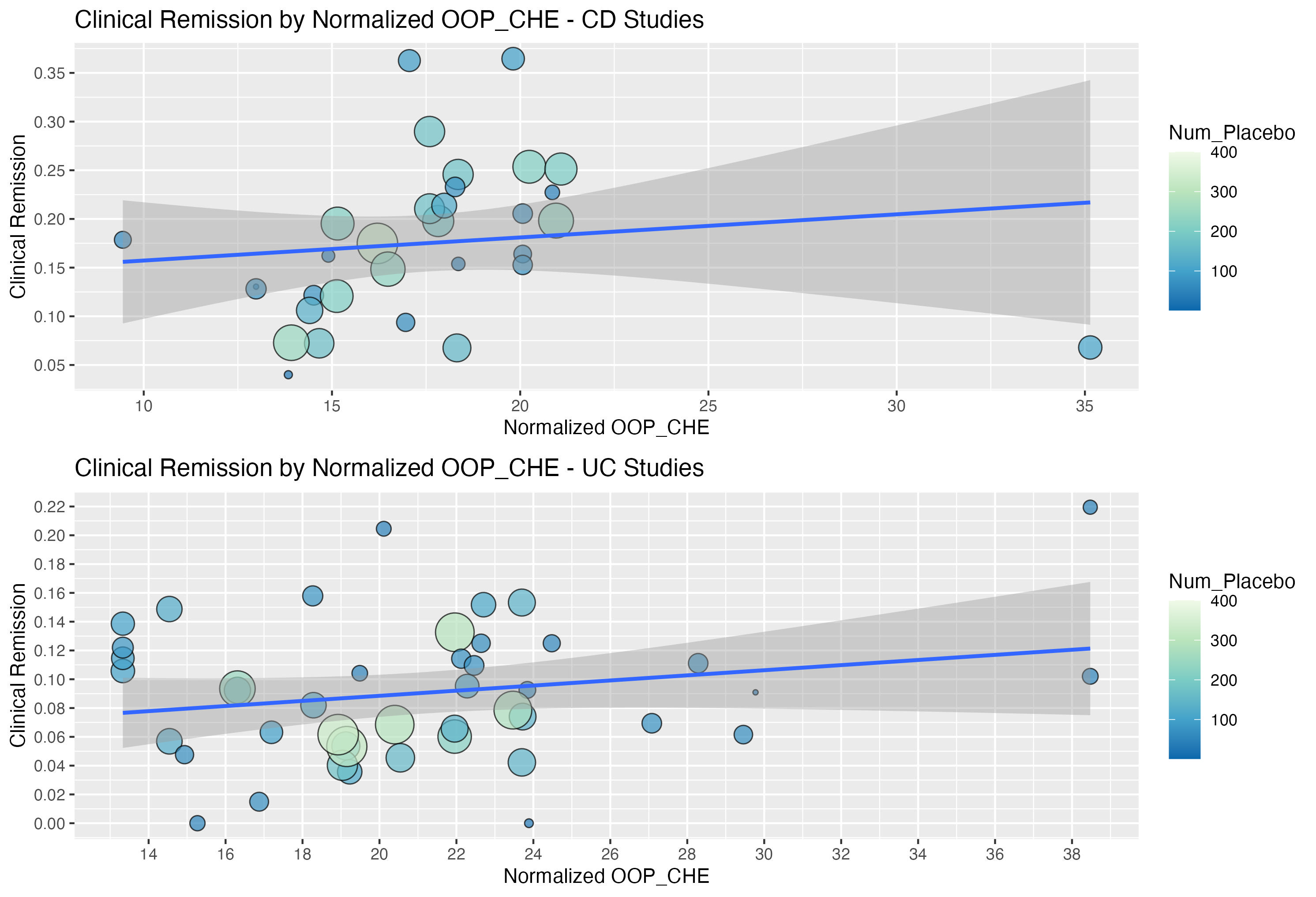

Supplement: jjag081_Supplementary_Data [file jjag081_supplementary_data.zip › Figure S8 - Clinical Response by Normalized OOP_CHE.jpeg]

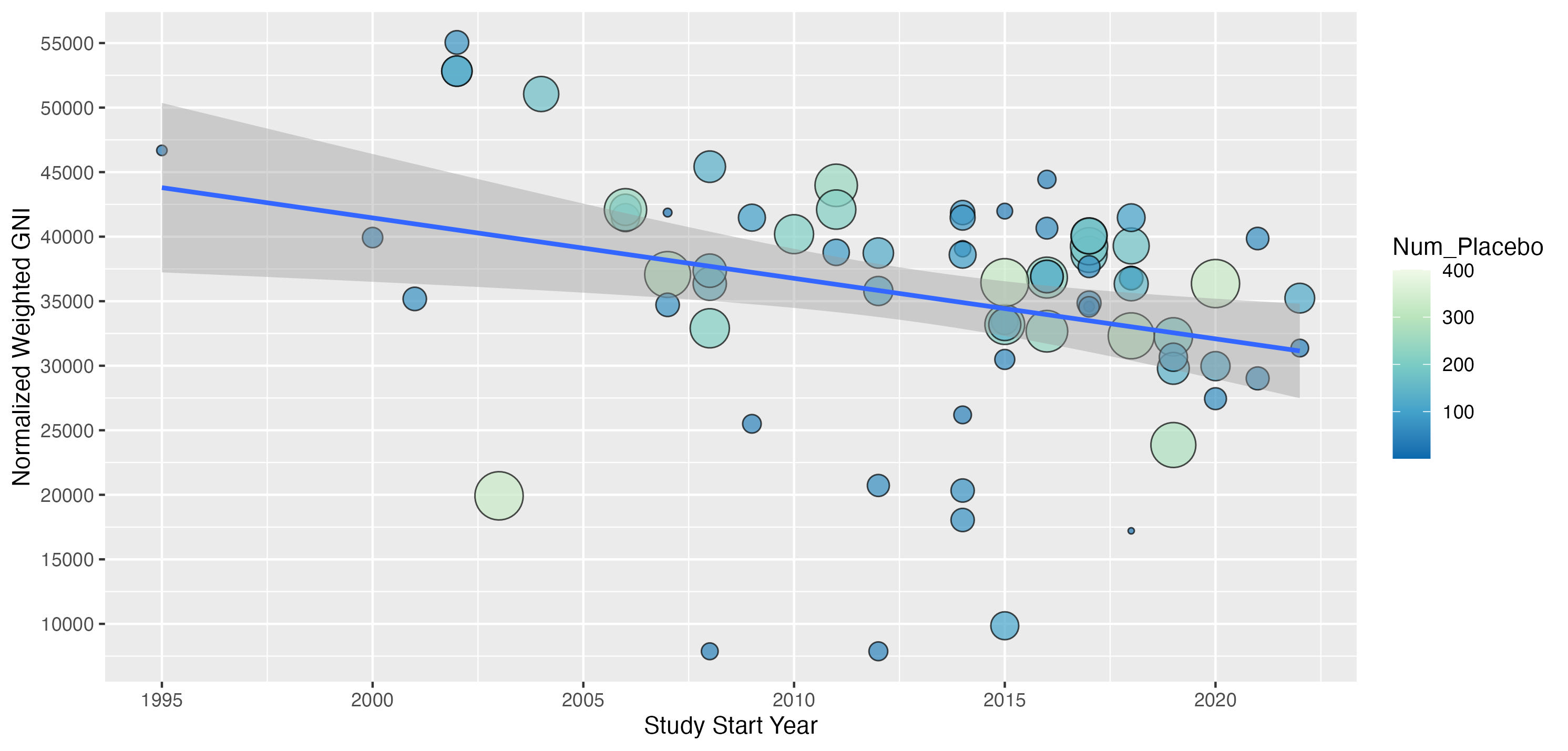

Supplement: jjag081_Supplementary_Data [file jjag081_supplementary_data.zip › Figure S1 - GNI by Year.jpeg]

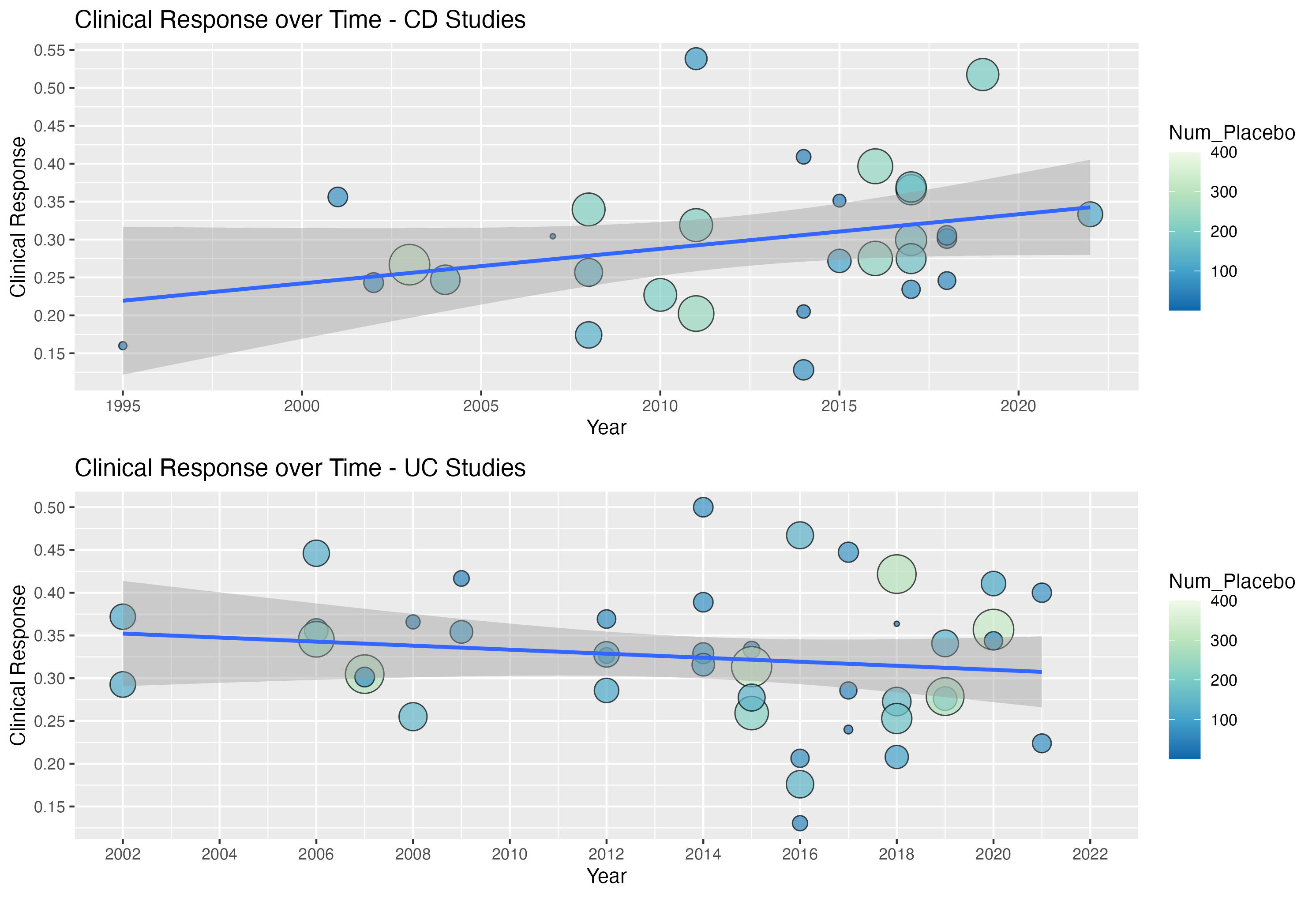

Supplement: jjag081_Supplementary_Data [file jjag081_supplementary_data.zip › Figure S2 - Clinical Response over time.jpeg]

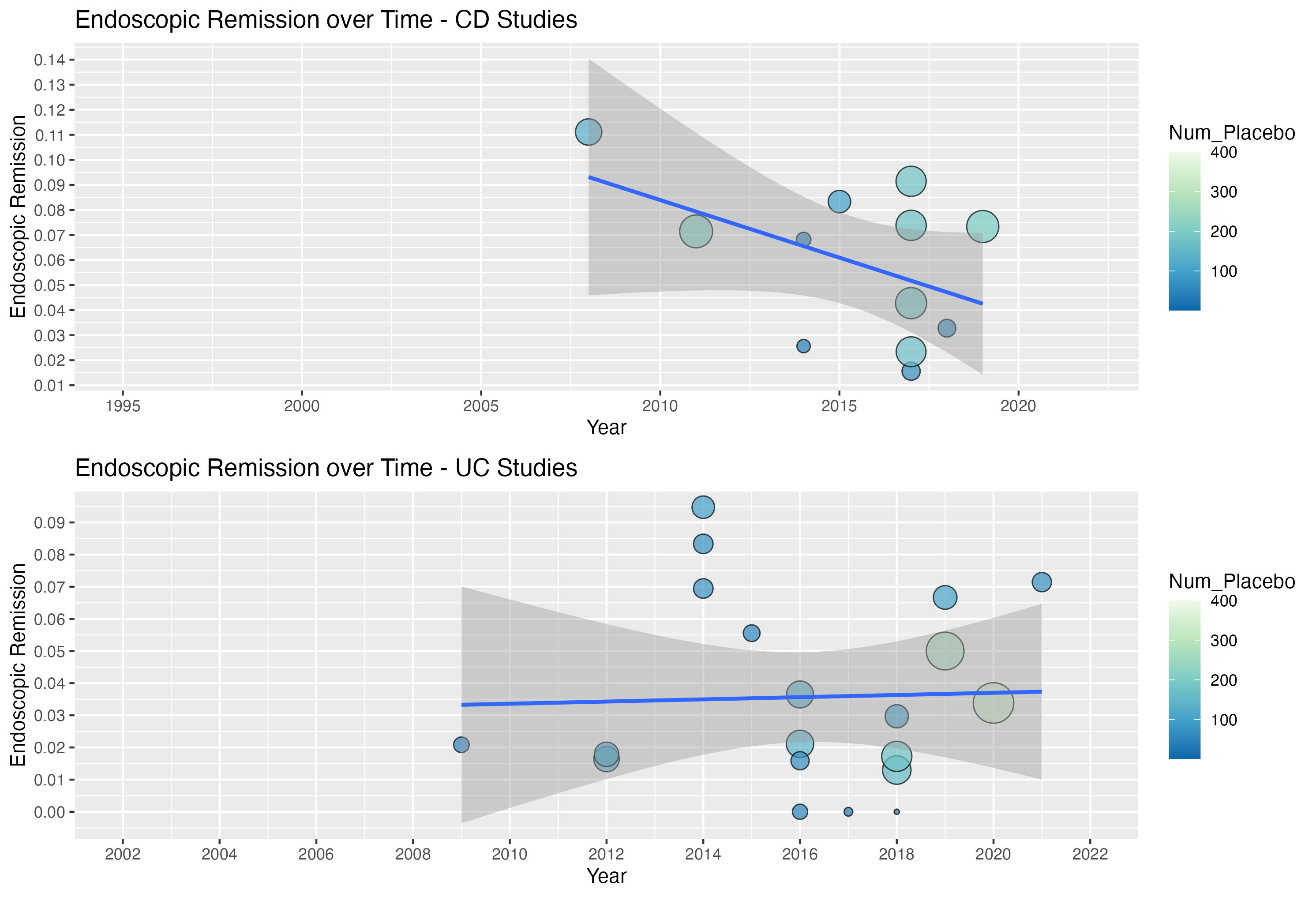

Supplement: jjag081_Supplementary_Data [file jjag081_supplementary_data.zip › Figure S3 - Endoscopic Remission over time.jpeg]

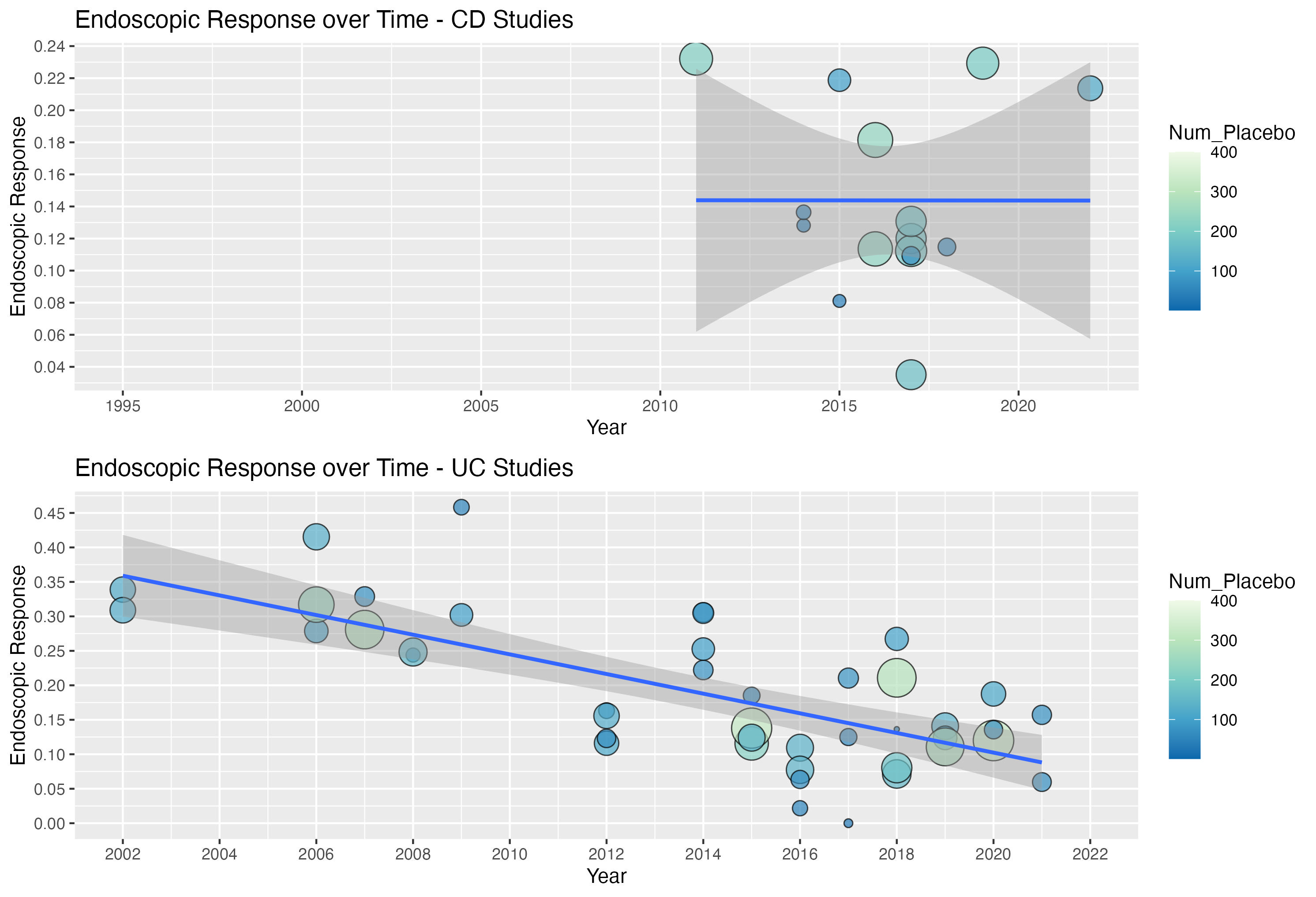

Supplement: jjag081_Supplementary_Data [file jjag081_supplementary_data.zip › Figure S4 - Endoscopic Response over time.jpeg]

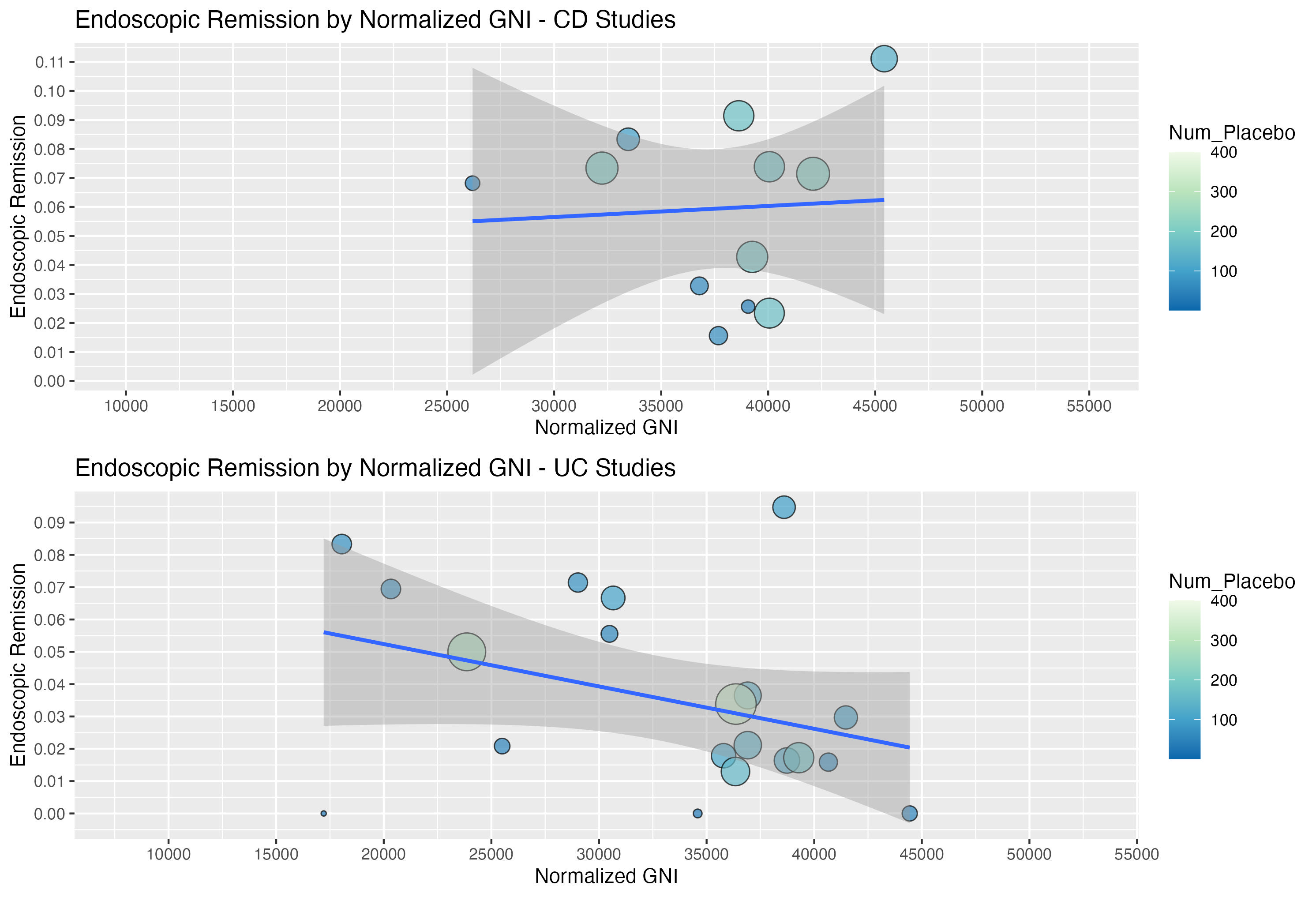

Supplement: jjag081_Supplementary_Data [file jjag081_supplementary_data.zip › Figure S5 - Endoscopic Remission by Normalized GNI.jpeg]

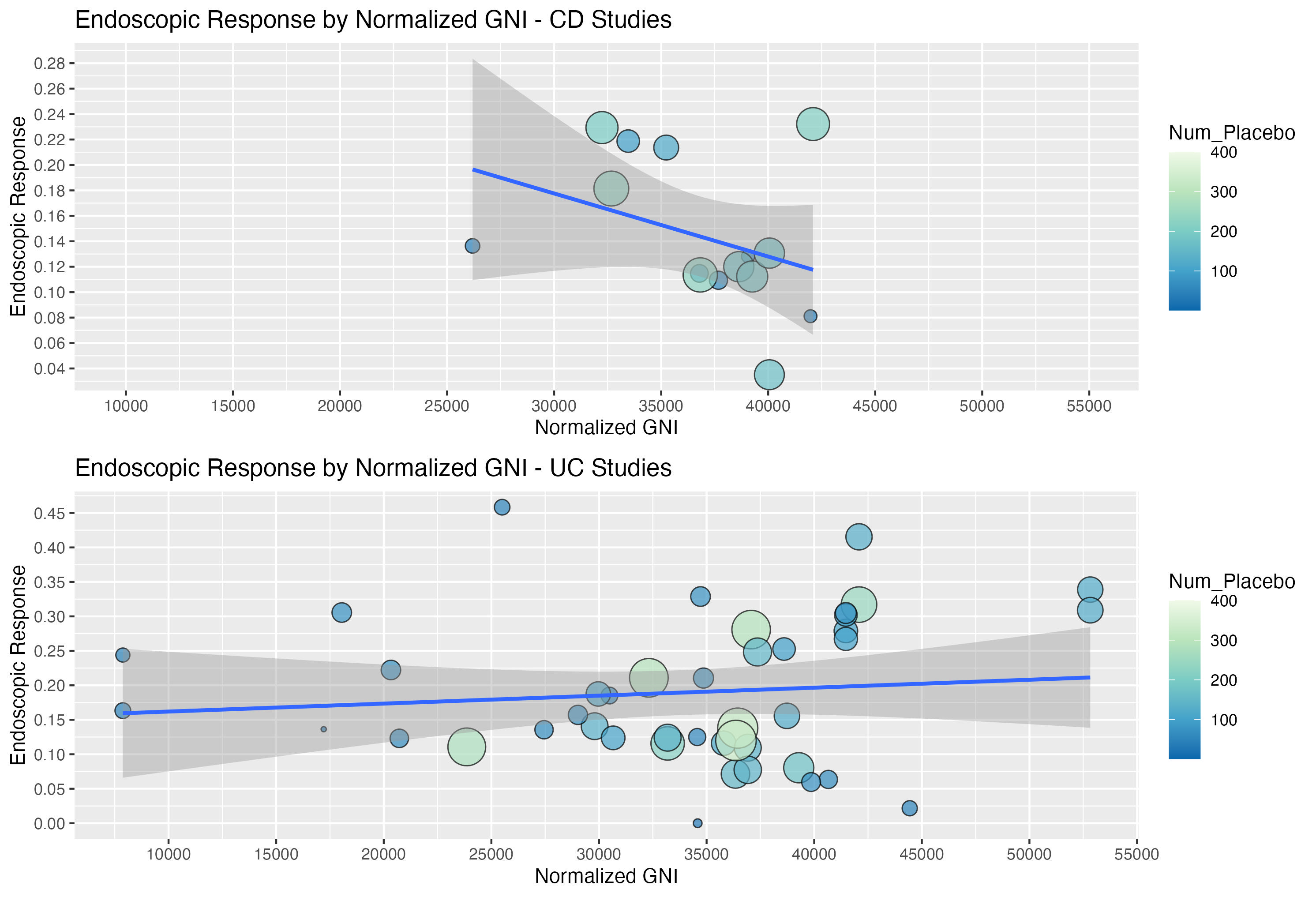

Supplement: jjag081_Supplementary_Data [file jjag081_supplementary_data.zip › Figure S6 - Endoscopic Response by Normalized GNI.jpeg]
